# Treatment of Hepatic Cystic Echinococcosis Patients with Clear Cell Renal Carcinoma: A Case Report

**DOI:** 10.1515/biol-2019-0073

**Published:** 2019-12-31

**Authors:** Bo Ran, Lujin Cheng, Lin Kang, Tuerganaili Aji, Tieming Jiang, Ruiqing Zhang, Qiang Guo, Hao Wen, Yingmei Shao, Hui Xiao

**Affiliations:** 1 School of Public Health, Xinjiang Medical University, No. 1, Xinjiang China; 2 Department of Hepatobiliary & Hydatid Disease, Digestive & Vascular Surgery Center, First Affiliated Hospital of Xinjiang Medical University, Urumqi, Xinjiang, 830054, China; 3 School of Public Health, Xinjiang Medical University, Urumqi, Xinjiang 830011, China; 4 Department of Stomatology, First Affiliated Hospital of Xinjiang Medical University, Urumqi, Xinjiang, 830011, China

**Keywords:** Echinococcosis, Clear cell renal carcinoma, liver

## Abstract

Human cystic echinococcosis is a zoonosis caused by the larval cestode *Echinococcus granulosus*. Clear cell renal carcinoma is the most common pathological type of renal cell carcinoma. Echinococcosis complicated with carcinoma is rarely reported. Here, we reported a female patient with echinococcal cyst of the liver accompanied with clear cell renal carcinoma. This 27-year-old woman was admitted for abdominal pain. The serological testing of hydatid cyst was positive and levels of tumor markers were within the normal range. The computed tomography and histological findings confirmed hepatic echinococcal cyst complicated with renal carcinoma of kidney. Preoperative liver function was grade A. The patient underwent pericystectomy of liver hydatid cyst and partial nephrectomy. No recurrence was found at 1 year of follow-up. Liver hydatid complicated with renal cell carcinoma is rare, which should be differentiated from liver metastasis of renal cancer. Surgical resection is the optimal treatment. This case may provide insight for the diagnosis and research on the co-occurrence of tumor and hydatid cyst.

## 1 Introduction

Cystic hydatid disease or cystic echinococcosis (CE) is a kind of parasitic zoonosis caused by the larva of the dog tapeworm *Echinococcus granulosus*. *Echinococcus granulosus* impacts both population health and animal production in Central Asia, the Mediterranean countries, and South America [1]. The most frequent site for the cystic lesions is liver, followed by lung, brain and other organs [2]. Surgery is the only curative treatment for CE [3].

Renal cell carcinoma (RCC) is the third most common tumor of the urinary system and accounts for approximately 2% to 3% of adult malignant tumors [4]. Approximately 18% of patients with RCC have metastasis at diagnosis [5]. Metastatic RCCs may occur in virtually all organ systems, but are mainly in the lungs, bones, and liver [6,7]. The mean survival time of patients with distant metastatic RCC is approximately 13 months [4]. Clear cell carcinoma is the most common pathological type of RCC and accounts for about 3% of adult malignant tumors [8]. The main treatment for renal clear cell carcinoma is surgery, including radical nephrectomy and partial nephrectomy, but the postoperative recurrence rate is 25% ~ 50% [9]. In addition, renal clear cell carcinoma is not sensitive to radiotherapy and chemotherapy [10].

Clinically, concomitant presence of hydatid cyst and tumor is very rare. Akgu et al reported only 2 patients with both tumor and Echinococcosis in a total of 2,086 tumor patients from 1990 to 2001 in Turkey [11]. Ali et al found only one patient with both acute leukemia and hepatococcosis in 1,200 patients with different blood diseases from 1985 to 2003 in Turkey [12]. In this case report, we presented a female patient with liver CE accompanied by renal clear cell carcinoma from Xinjiang, China.

## 2 Case report

**Figure 1:**
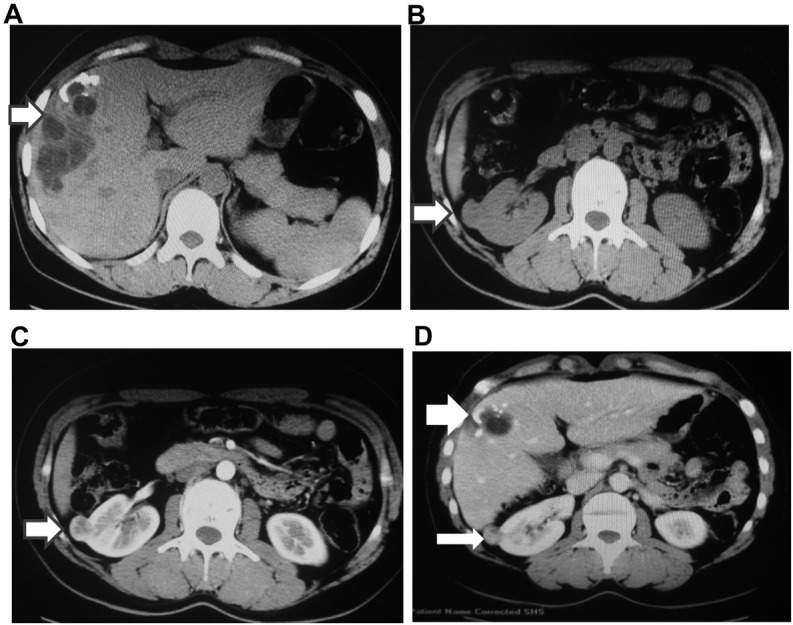
Photographs of computed tomography (CT) of the liver and kidney. A. CT-scan (plain) showing the parasitic cystic lesion in the right hepatic lobe (indicated by arrow). B. CT-scan (plain) showing the tumor in the right kidney (indicated by arrow). C. The lesion of kidney was significantly enhanced after contrast enhancement (indicated by arrow). D. CT-scan showing carcinoma of kidney (thin arrow) complicated with echinococcal cyst of the liver (thick arrow).

A 27-year-old female patient was admitted to the First Affiliated Hospital of Xinjiang Medical University for recurrent abdominal pain. This patient came from pastoral areas and had a history of contact with dogs and sheep. She had no associated history of vomiting, fever, jaundice or weight loss. Physical examination showed mild tenderness in the right upper quadrant of abdomen. The results of liver function, kidney function and coagulation function were normal. The levels of tumor markers, including CA 125, alpha fetoprotein, carcinoembryonic antigen and CA19–9 were within the normal ranges. The dot immunogold filtration assay showed that the antibodies of EgCF, EgP, EgB were positive and the Em2 antibody was negative. The abdominal ultrasound showed a cystic mass (about 6 cm in diameter) in liver and a solid mass (about 2 cm in diameter) in kidney. Computed tomography (CT) confirmed a 6x6cm lesion in the right hepatic lobe, with irregular margins, unclear boundary, calcification of the wall and heterogeneous density (Fig. 1A), as well as a mass with a size of 2x2 cm in the right kidney (Fig. 1B). Most of the lesions protruded from the kidney, and the lesion of kidney was significantly enhanced after contrast enhancement (Fig. 1C). Finally, she was diagnosed with liver Echinococcal cyst accompanied with clear cell renal carcinoma (Fig. 1D).

**Figure 2:**
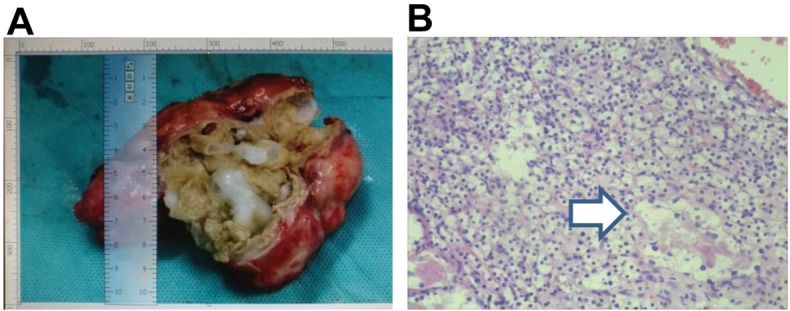
The patient was diagnosed as hepatic cystic echinococcosis accompanied with clear cell renal carcinoma. A. Multiple cysts were seen in the open hydatid exocapsule. B. H&E staining of kidney lesion (indicated by arrow; magnification, X400).

Then, pericystectomy of liver hydatid cyst and partial nephrectomy was performed. The typical hydatid cyst was observed when the liver lesion was opened (Fig. 2A). The hematoxylin and eosin staining of renal mass revealed large polygonal cells with clear cytoplasm, uniform round nuclei, and inconspicuous nucleoli, surrounded by a fibrous capsule (Fig. 2B). Immunohistochemistry showed positive staining for Ck7, P504S, CAM 5.2, CD10, EMA, CA9, Vim, Pax-8 and negative for CD117, which were typical findings of clear cell renal carcinoma. Albendazole was administered postoperatively for 3 months at a dose of 15mg/kg per day. The patient recovered well after the operation, and no recurrence was found at the one year follow-up.

**Informed consent**: Informed consent has been obtained from all individuals included in this study.

**Ethical approval**: The research related to human use has been complied with all the relevant national regulations, institutional policies and in accordance the tenets of the Helsinki Declaration, and has been approved by the ethics review board of Xinjiang Medical University.

## 3 Discussion

CE is common in certain geographical areas (Africa, Middle East, Mediterranean Sea, South America, Australia, New Zealand), but its frequency tends to be decreased with the improvement of sanitary conditions and the development of effective management procedures [13]. The disease predominantly affects the liver (70%), mainly the right lobe, and the solitary cyst is found in 80% of cases [14]. The diagnosis can be established by CT scan, in which the hepatic CE are water-like density of cystic lesions, and calcification of the cyst wall and daughter cyst or collapsed inner cyst can be seen [14,15].

Surgery remains the main treatment method for CE [16]. Basically, there are two open surgical methods termed as conservative surgical procedure and radical surgical procedure [3]. The conservative surgical procedure (including simple drainage, partial cystopericystectomy, and subtotal pericystectomy) is a tissue-sparing technique, which only removes the parasitic tissues with the entire or sometimes part of the pericyst (host tissue layer) left in situ. However, this procedure has some postoperative complications such as biliary fistula, a residual cavity, and cavity suppuration, which remarkably impacts the recovery of patients. The radical surgical procedure (including total pericystectomy and liver resection) may overcome these complications. In this report, total pericystectomy was applied and a good result was achieved. Thus, we propose that total pericystectomy is the first choice for CE.

RCC is a malignant tumor that often metastasizes to lung, liver, bone and brain at early stage, and approximately 33% of RCC patients has metastatic masses, in which liver metastasis accounts for about 20% [17]. McKay et al. reported that the presence of bone and liver metastases had a negative impact on survival [18]. In this case, the mass in the liver had inhomogeneous density and irregular margins. This is different from the typical finding of liver CE imaging. Since the incidence of hydatid tumors is extremely low, we cannot rule out the possibility of liver metastasis of renal cancer before surgery. During the operation, we found that the irregular performance of hepatic lesion was resulted from the protrusion of the hydatid cyst, and the uneven CT density was caused by partial necrosis of the cyst contents.

The co-occurrence of hydatid cyst and tumor is very rare. To date, the relationship between these two diseases is still not accurately defined. Some reports show that Echinococcosis can inhibit tumor growth. For example, In 1980, van Knapen et al. first proposed that infection with hydatid could inhibit tumor growth [19]. In 2012, Yousofi et al found that Echinococcosis had an inhibitory effect on the growth of fibrosarcoma cells in mice [20]. In 2013, Karadayi et al found the serum of hydatid patients inhibited the growth of small-cell lung cancer in animal experiments [21]. However, the effect of CE on renal clear cell carcinoma is still unclear. Whether infection by Echinococcus cyst could increase or decrease the risk of renal clear cell carcinoma needs to be investigated in future studies with more cases.

In summary, we reported a rare case of hepatic Echinococcal cyst coexisting with clear cell renal carcinoma. Our study indicates that it is necessary to differentiate liver metastasis of renal carcinoma from liver CE in clinical diagnosis. This case may also provide scientific value for the research on tumor and hydatid cyst.
